# Lipase mimetic cyclodextrins†

**DOI:** 10.1039/d0sc05711h

**Published:** 2020-11-19

**Authors:** Youngjun Lee, Neal K. Devaraj

**Affiliations:** Department of Chemistry and Biochemistry, University of California San Diego, 9500 Gilman Drive La Jolla CA 92093 USA ndevaraj@ucsd.edu

## Abstract

Glycerophospholipids (GPLs) perform numerous essential functions in biology, including forming key structural components of cellular membranes and acting as secondary messengers in signaling pathways. Developing biomimetic molecular devices that can detect specific GPLs would enable modulation of GPL-related processes. However, the compositional diversity of GPLs, combined with their hydrophobic nature, has made it challenging to develop synthetic scaffolds that can react with specific lipid species. By taking advantage of the host–guest chemistry of cyclodextrins, we have engineered a molecular device that can selectively hydrolyze GPLs under physiologically relevant conditions. A chemically modified α-cyclodextrin bearing amine functional groups was shown to hydrolyze lyso-GPLs, generating free fatty acids. Lyso-GPLs are preferentially hydrolyzed when part of a mixture of GPL lipid species, and reaction efficiency was dependent on lyso-GPL chemical structure. These findings lay the groundwork for the development of molecular devices capable of specifically manipulating lipid-related processes in living systems.

## Abstract 2

Lipase mimetic cyclodextrins (CDs) can selectively hydrolyze native lyso-glycerophospholipids (GPLs) under physiologically relevant conditions.

## Graphical abstract

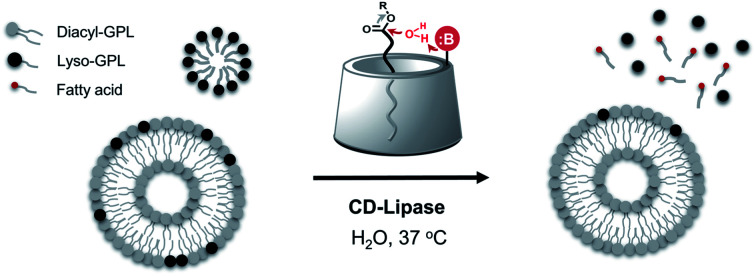

## Introduction

Glycerophospholipids (GPLs) are essential amphiphilic molecules found in all living organisms.^1^ Di-acylated GPLs play a major structural role in cellular lipid membranes, which separate internal cytoplasmic components from the extracellular environment.^2,3^ Mono acylated (lyso-) GPLs are metabolic intermediates of diacyl GPLs that are detected in cells but are not directly involved in bilayer formation, instead playing roles in regulation of membrane fluidity and permeability.^4^ Lyso-GPLs also function as bioactive signaling molecules, involved in angiogenesis,^5^ inflammation,^6^ and they have been implicated in human diseases, such as cancer.^7^ Thus, selective modulation of lyso-GPLs would be highly desirable for manipulating *in vivo* GPL levels and as a tool for determining the functions of lyso-GPLs in lipid signaling pathways.^8^ While enzymatic approaches have been studied for such purposes,^9^ we sought to explore whether synthetic scaffolds could be responsive to lyso-GPLs. Synthetic scaffolds would have advantages compared to enzymes with respect to cost, size, and stability. Efforts to develop small-molecule lyso-GPL modulators that work under physiological conditions may ultimately lead to the development of novel therapeutics for lipid metabolism disorders.^10^

Cyclodextrins (CDs) are water-soluble cyclic oligosaccharides produced by bacterial cyclodextrin glucanotransferase.^11^ The host–guest chemistry of CDs, along with their amenability to synthetic modification,^12^ has faciliated the fabrication of enzyme mimetic CDs, such as glycosidases,^13^ esterases,^14^ and proteases.^15^ Randomly methylated α- or β-CD (RMαCD or RMβCD) can be utilized as hosts for membrane lipid species such as GPLs and cholesterols.^16^ Applications include introducing exogenous phospholipids into cultured cells,^17^ generating asymmetric phospholipid membranes in vesicles,^18^ or the extraction and transfer cholesterols from a mixture of cellular lipids.^19^ However, most previous studies with RMCDs have utilized commonly available CDs as lipid absorbers and there are currently no reports of CDs capable of reacting with specific native lipid species. Given previous work on the use of CDs as enzyme mimetics,^20^ we hypothesized that modified CDs might act as lipase mimetics by binding to and positioning reactive functional groups in close proximity to hydrolytically susceptible linkages in the lipid substrate. Herein, we report a CD-based host that can selectively hydrolyze lyso-GPLs under physiologically relevant conditions.

## Results and discussion

### Preparation of CD-based lipase mimetics

**Fig. 1:**
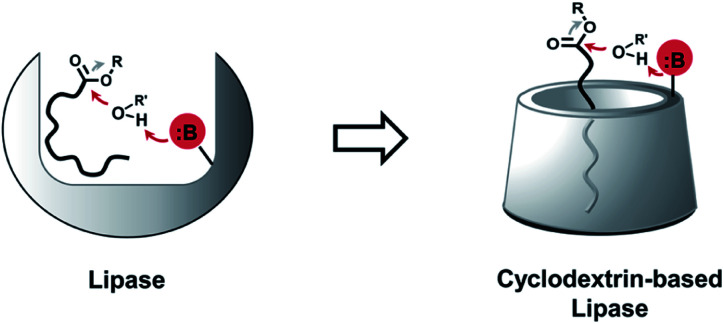
Schematic representation showing the development of lipase mimetic cyclodextrins (CDs) inspired by the mechanism of base promoted hydrolysis in the active site of natural lipases [B: base, R: alkyl group, R′: alkyl group or proton].

Phospholipases cleave ester bonds of GPLs, liberating their fatty acid and glycerol components.^21^ To mediate hydrolysis, enzyme active sites generally contain a histidine base that increases the nucleophilicity of a nearby serine hydroxyl group (Fig. 1, left), which attacks the ester of GPL substrates. Inspired by the enzymatic mechanism, we envisioned the development of modified CDs that could not only bind to GPLs, but also induce hydrolysis of native esters under ambient aqueous conditions. To explore this idea, imidazole or amine modified α- and β-CDs were synthesized (Table 1). We hypothesized that the introduction of basic moieties would lead to enhanced local nucleophilic reactivity by activating the surrounding water molecules or the neighboring hydroxyl groups on CDs (Fig. 1, right). Ester-containing lipid molecules confined by the host–guest interactions would be selectively cleaved. Previous studies suggest that the smaller hydrophobic cavity of α- and β-CD (4.7–5.3 and 6.0–6.5 Å, respectively) would serve as more efficient hosts for aliphatic hydrocarbons than that of larger γ-CD (7.5–8.3 Å).^16,22^ To test these ideas, we prepared chemically modified α- and β-CD derivatives with mono or persubstituted basic functional groups to identify potential lipase mimics that can react with native lipid substrates. Adapting previously published procedures,^23–25^ substitution reactions with imidazole afforded A3, B3, and B4 in good yield and purity (Table 1), whereas substitution with azide followed by Staudinger reduction generated A5, A6, B5, and B6 (for details refer to ESI†). Unmodified (A1 and B1) and randomly methylated (A2 and B2) CDs were included as control CDs that lack basic functionalities.

**Table 1:** Chemical structure and list of CD derivatives used in this study

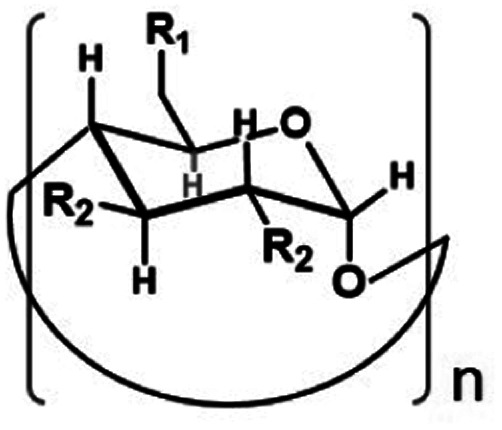					
αCD (*n* = 6)	R_1_	R_2_	βCD (*n* = 7)	R_1_	R_2_
A1	OH	OH	B1	OH	OH
A2	OH/OMe	OH/OMe	B2	OH/OMe	OH/OMe
A3	Imidazole^a^	OH	B3	Imidazole^a^	OH
A4	Imidazole	OH	B4	Imidazole	OH
A5	NH_2_^a^	OH	B5	NH_2_^a^	OH
A6	NH_2_	OH	B6	NH_2_	OH

a Monosubstituted.

### Evaluation of lipase mimetic activity

**Fig. 2:**
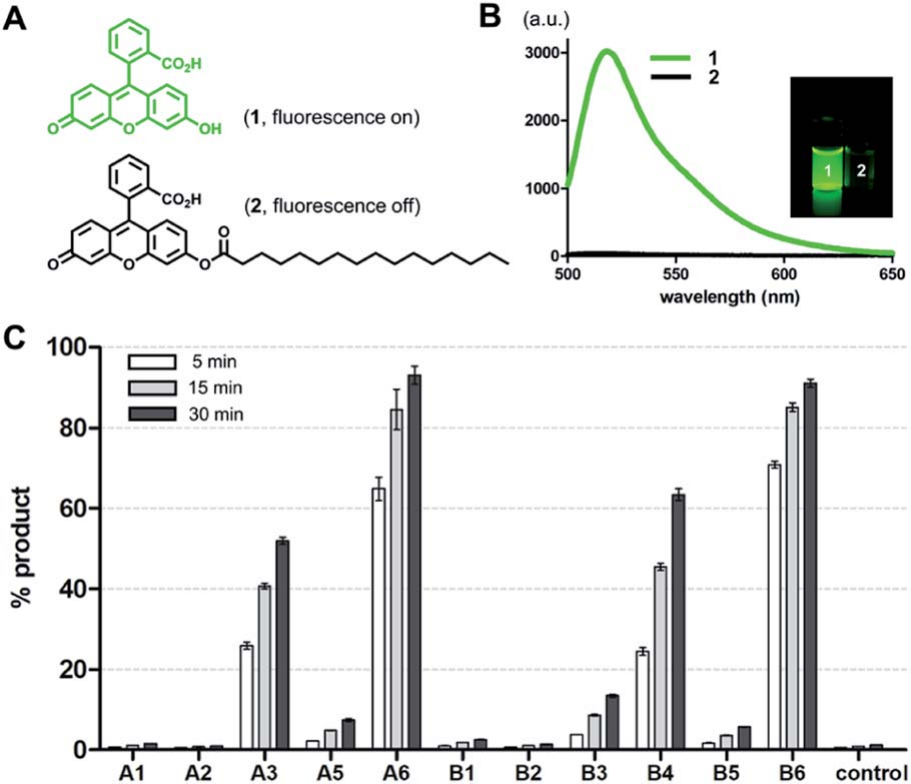
Evaluation of lipase mimetic activity for modified CDs using a reaction-based fluorogenic reporter. (A) Chemical structures of fluorescein (1) and its acylated fluorogenic substrate (2). (B) Fluorescence emission spectra of 1 and 2 excited at 480 nm. An inset photograph compares visible fluorescence of 1 and 2 under a handheld UV lamp. (C) The percent yield of compound 1 generated from the reaction between compound 2 (1 mM) and the corresponding CD derivatives** (1 mM) after 5, 15, and 30 min. Reactions were performed in 0.02 M bicine (pH 8.0) with 1% DMSO (v/v) at 37 °C. Error bars represent standard deviation from *n* = 3 replicates. **A4 was not tested due to the presence of inseparable impurities.

To screen the hydrolytic activity of the chemically modified CDs in a quantitative manner, we synthesized a fluorogenic lipid substrate with a cleavable ester bond. Previous work has demonstrated that a fluorophore having an acylated exocyclic hydroxyl group on a conjugated pi electron system can serve as a reaction-based reporter that responds to cleavage of the ester linkage.^26^ We selected fluorescein as the reporter headgroup (Fig. 2A, 1), and acylated it with palmitoyl chloride to obtain compound 2 as a model fluorogenic lipid substrate. Considering the cavity sizes of α-CD (4.7–5.3 Å) and β-CD (6.0–6.5 Å),^22^ it is expected that the major interactions between the CDs and compound 2 takes place on the palmitoyl lipid tail, instead of the larger charged fluorescein head group (Fig. S1†). Acylated compound 2 exhibited 80-fold less fluorescence emission compared to that of the hydrolysis product compound 1 (Fig. 2B). Using fluorogenic substrate 2, we evaluated the lipase mimetic activity of A1–A6 and B1–B6 in pH 8 buffer solution for 1 hour at 37 °C. Unmodified CDs (A1 and B1) and RMCDs (A2 and B2) lacking basic functional groups were unable to induce the ester cleavage reaction (Fig. 2C). In contrast, the modified CDs generated hydrolysis products. The persubstituted CDs displayed increased activity compared to the monosubstituted CDs (A6*vs.*A5, B4*vs.*B3, and B6*vs.*B5), and the peramino-CDs, A6 and B6, showed the greatest lipase mimetic activity. Additionally, A6 exhibited greater water solubility when compared to B6, making it the most suitable lipase mimetic in aqueous solutions. As a control, we compared the activity of A6 to 1, 5, or 10 mM dimethylamine or imidazole, which further validated the superior activity of peraminated A6 over isolated basic functional groups (Fig. S2†). A6 was therefore selected for mimicking lipase activity under ambient aqueous conditions using natural lipid substrates.

### Lipase activity towards native GPLs

**Fig. 3:**
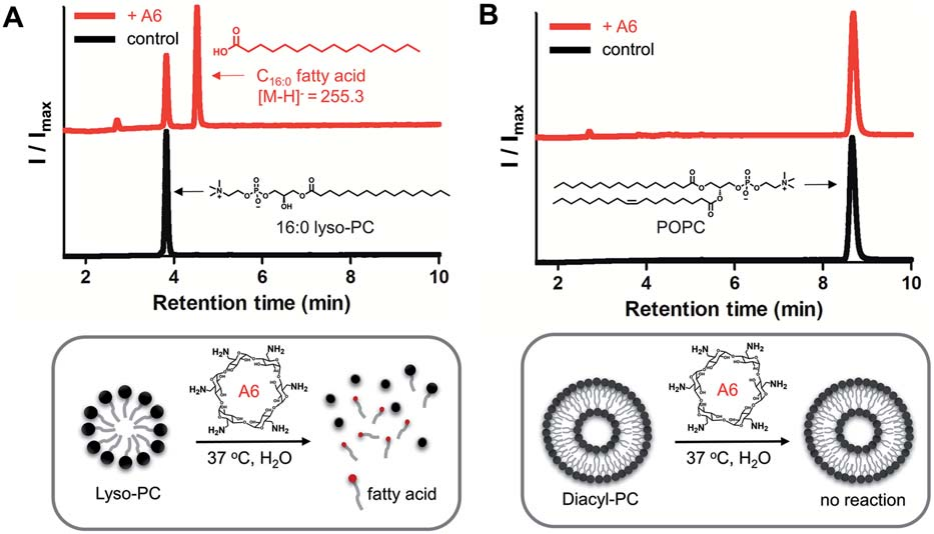
Reaction results of A6-mediated hydrolysis towards native lyso- and diacyl-GPLs. (A) Reaction between 16 : 0 lyso-PC (1 mM) and A6 (4 mM, upper red line) or control (lower black line), (B) reaction between POPC (1 mM) and A6 (4 mM, upper red line) or control (lower black line). Reactions were conducted in 0.02 M bicine buffer (pH 8.0) for 3 days at 37 °C. ELSD peaks generated from reactions were verified by MS spectrometry. 16 : 0 lyso-PC: 1-palmitoyl-2-hydroxy-*sn*-glycero-3-phosphocholine, POPC: 1-palmitoyl-2-oleoyl-*glycero*-3-phosphocholine.

To demonstrate lipase mimetic activity on native ester bonds, we investigated the reaction between A6 and various choline containing GPLs. Fatty acid products were observed from the reaction between A6 and single-chain lyso-GPLs, such as 1-palmitoyl-2-hydroxy-*sn*-glycero-3-phosphocholine (16 : 0 lyso-PC) or 1-oleoyl-2-hydroxy-*sn*-glycero-3-phosphocholine (18 : 1 lyso-PC) in pH 8 buffer solution for 3 days at 37 °C (Fig. 3A and S3A†). This demonstrates that A6 induces hydrolysis of the ester linkage of lyso-GPLs, generating their component fatty acids as hydrolysis products. In contrast, under the same reaction conditions, when A6 was added to diacyl-GPLs such as 1-palmitoyl-2-oleoyl-*glycero*-3-phosphocholine (POPC), 1,2-dipalmitoyl-*sn*-glycero-3-phosphocholine (DPPC), or 1,2-dioleoyl-*sn*-glycero-3-phosphocholine (DOPC), we did not observe any products by HPLC (Fig. 3B, S3B and C†). These results could be explained by a combination of the solubility differences between the diacyl-GPLs and lyso-GPLs and that A6 has a stronger preference for single-chain GPLs than diacyl-GPLs, presumably due to the selectivity of the CD host. We observed that diacyl-GPLs were able to self-assemble into stable vesicles, even in the presence of A6 (Fig. S4†), suggesting that diacyl-GPLs in vesicles were unavailable to interact with the thermodynamically less favorable CD host. In contrast, lyso-GPLs typically pack to form significantly less stable micelles, with the result that A6 is more likely to bind to single-chain GPLs *versus* diacyl-GPLs. Using 16 : 0 lyso-PC, we further optimized the lipid to CD ratio (Fig. S5†), and observed quantitative hydrolysis of 16 : 0 lyso-PC, albeit at an elevated temperature of 50 °C. Under the optimized conditions, A6 exhibited significant lipase mimetic activity between pH 7–9 (Fig. S6†).

**Fig. 4:**
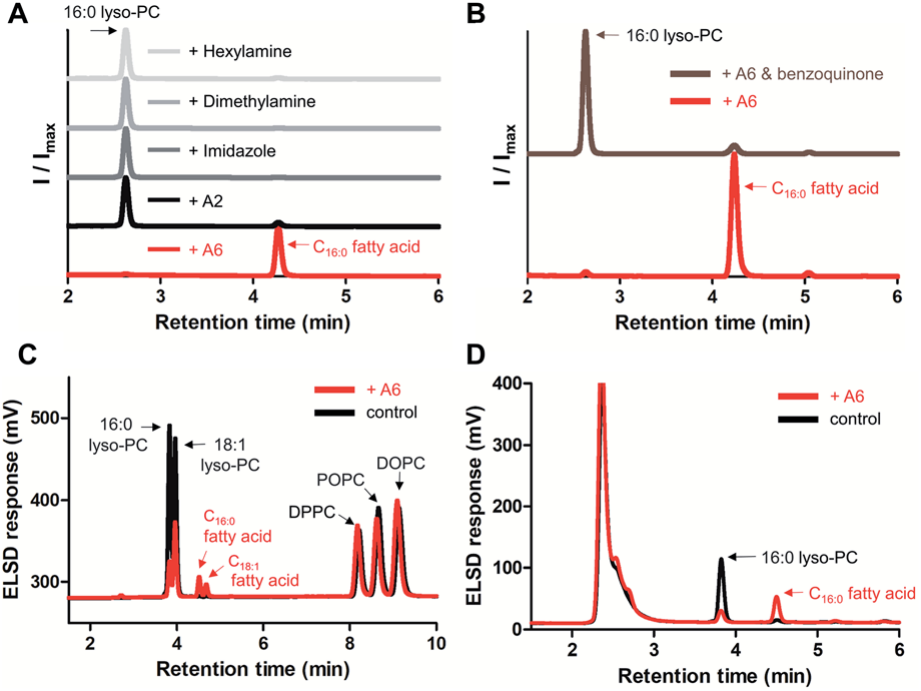
Characterization of A6 as a lipase mimetic for the hydrolysis of lyso-GPLs. (A) Hydrolytic activity of A6, A2, imidazole, dimethylamine, and hexylamine (4 mM each) towards 16 : 0 lyso-PC (1 mM), (B) inhibition of A6 using 1,4-benzoquinone (10 mM) as a competitive guest against 16 : 0 lyso-PC (1 mM), and (C) selective hydrolysis of 16 : 0 lyso-PC (1 mM) and 18 : 1 lyso-PC (1 mM) from a combined mixture of mono and diacyl-GPLs (1 mM each), (D) hydrolytic activity of A6 (4 mM) toward 16 : 0 lyso-PC (1 mM) in 10% FBS supplemented DMEM. Reactions were conducted in 0.02 M bicine buffer (pH 8.0) for 3 days at 50 °C (A and B) or 37 °C (C) or in 10% FBS supplemented DMEM for 3 days at 37 °C (D). ELSD peaks generated from reactions were verified by MS spectrometry. 18 : 1 lyso-PC: 1-oleoyl-2-hydroxy-*sn*-glycero-3-phosphocholine, DOPC: 1,2-dioleoyl-*sn*-glycero-3-phosphocholine, DPPC: 1,2-dipalmitoyl-*sn*-glycero-3-phosphocholine.

Subsequently, we compared the hydrolytic reactivity of A6 with the randomly methylated CD A2, which is known to be an efficient solubilizing agent for single-chain lipids,^14^ as well as with other nucleophiles, such as imidazole, dimethylamine, and hexylamine. Only A6 generated a clear hydrolytic product in pH 8 buffer over 3 days at 50 °C (Fig. 4A). To determine if the CD cavity is required to achieve lipase mimetic activity, we added excess 1,4-benzoquinone to the reaction mixture, a known competitive guest of CDs.^27,28^ When A6 was in the presence of 1,4-benzoquinone, hydrolysis was substantially reduced (Fig. 4B), providing evidence that the host–guest interaction is required for the A6-induced hydrolysis reaction.

We analyzed the selectivity of GPL hydrolysis by treating A6 with a combined mixture of lyso-GPLs (16 : 0 lyso-PC and 18 : 1 lyso-PC) and diacyl-GPLs (POPC, DPPC, and DOPC). In pH 8 buffer at 37 °C, we confirmed that diacyl-GPLs were not affected by A6 (right side of Fig. 4C) over 3 days, but both lyso-GPLs from the lipid mixture were selectively hydrolyzed (left side of Fig. 4C). Under microscopy, we observed multiple vesicles in the reaction mixture (Fig. S7†), indicating that A6 can react selectively with lyso-PCs in the presence of complex lipid membranes. In addition, we observed hydrolytic activity of A6 in Dulbecco's Modified Eagle Medium (DMEM) with 10% fetal bovine serum (FBS), a cell culture medium supplemented with various nutrients such as growth factors, lipids and hormones (Fig. 4D).^29^ This result suggests the usefulness of A6 as a selective lipase mimetic in more complex physiologically relevant settings.

### Hydrolytic efficiency depending on the GPL structures

**Fig. 5:**
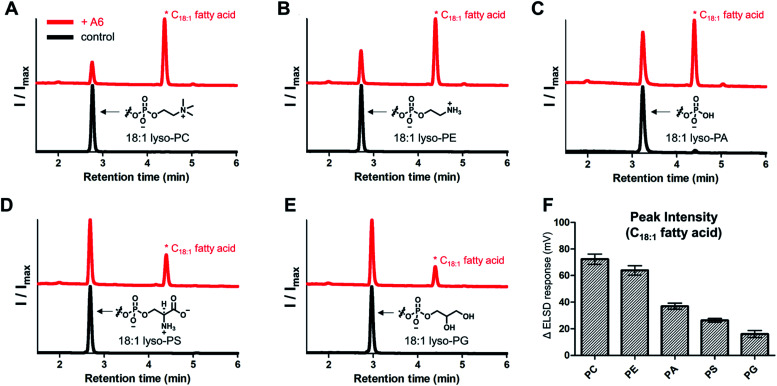
A6-Induced hydrolytic activity for 18 : 1 lyso-GPLs with different polar headgroups. Normalized HPLC-ELSD traces of the reaction between A6 and (A) 18 : 1 lyso-PC, (B) 18 : 1 lyso-PE, (C) 18 : 1 lyso-PA, (D) 18 : 1 lyso-PS, and (E) 18 : 1 lyso-PG (upper red lines) or controls (lower black lines). (F) ELSD intensity of the corresponding hydrolyzed products (C18 : 1 fatty acid) from spectra of (A–E). Error bars represent standard deviation from *n* = 6 replicates. All reactions were performed in 0.02 M bicine buffer (pH 8.0) at 50 °C for 3 days. The concentrations of lyso-GPLs and A6 were 1 and 4 mM, respectively.

To evaluate the range of substrates hydrolyzed by the lipase mimetic activity, we assessed A6 activity on various lyso-GPLs. 18 : 1 lyso-GPLs with differing polar headgroups were mixed with A6 in pH 8 buffer solution for 3 days at 50 °C. A6, in addition to hydrolyzing lyso-GPL with a PC headgroup, was also effective at hydrolyzing lyso-GPLs bearing phosphoethanolamine (PE), phosphatidic acid (PA), phosphoserine (PS), and phosphatidylglycerol (PG) headgroups (Fig. 5A–E). Lyso-GPLs bearing zwitterionic-type headgroups having a net charge of zero (PC and PE) were the most susceptible to hydrolysis by A6, while reduced activity was observed with lyso-GPLs bearing negatively charged headgroups (PA, PS and PG) (Fig. 5F). These results suggest that the dominant driving forces of CD-mediated lipid hydrolysis are hydrophobic interactions between the acyl chains and CD host, rather than electrostatic interactions between the negatively charged phosphate groups and the positively charged basic amine groups on A6. In terms of acyl chain structure, A6 showed clear hydrolytic activity on lyso-GPLs containing C12–C18 acyl chains (Fig. S8†) in pH 8 buffer solution for 3 days at 50 °C. A6 exhibited greater hydrolytic activity on the saturated 18 : 0 lyso-PC compared to the unsaturated 18 : 1 lyso-PC at 37 °C (Fig. S9†). This result suggests that CD-based biomimetics could be developed that can differentiate between lipids based on their degree of saturation.

## Conclusions

In conclusion, we have developed a CD-based lipase mimetic that can hydrolyze native lyso-GPLs in a selective manner. Through the combined effect of host–guest chemistry and base functionality, we have shown that chemically modified per-6-amino-α-CD A6 is capable of hydrolyzing the native ester linkage of lyso-GPLs under physiologically relevant conditions. Further studies on CD-based biomimetics will be geared toward enhancing the reactivity and selectivity for native lipids, possibly through further synthetic modification of CDs. For example, di- or tri-substitution of cooperative reaction units, incorporating molecular recognition motifs, or covalent dimerization of functional CDs could lead to improved reactivity and binding affinity towards native lipids.

## Conflicts of interest

There are no conflicts to declare.

## Supplementary Material

SC-012-D0SC05711H-s001
