# Supplementary material for: Lipase mimetic cyclodextrins
Source: Chem Sci. 2020 Nov 19;12(3):1090–4. doi: 10.1039/d0sc05711h (PMC8179069; doi:10.1039/d0sc05711h)
Supplement: SC-012-D0SC05711H-s001 [file SC-012-D0SC05711H-s001.pdf]

Electronic Supplementary Material (ESI) for Chemical Science.
This journal is © The Royal Society of Chemistry 2020

Electronic Supplementary Information for

Lipase mimetic cyclodextrins

*Youngjun Lee and Neal K. Devaraj**

Department of Chemistry and Biochemistry, University of California, San Diego,

E-mail: ndevaraj@ucsd.edu

Table of Contents

1	General Information	S2
2	Experimental Procedure	S3
3	Synthetic Procedure and Characterization of New Compounds	S4–S5
4	Supporting Figures	S6–S14
5	NMR spectra	S15–S18
6	References	S19

1. General Information

a) Materials

1-lauroyl-2-hydroxy-sn-glycero-3-phosphocholine (12:0 lyso-PC), 1-myristoyl-2-hydroxy-sn-glycero-3-phosphocholine (14:0 lyso-PC), 1-palmitoyl-2-hydroxy-sn-glycero-3-phosphocholine (16:0 lyso-PC), 1-stearoyl-2-hydroxy-sn-glycero-3-phosphocholine (18:0 lyso-PC), 1-oleoyl-2-hydroxy-sn-glycero-3-phosphocholine (18:1 lyso-PC), 1,2-dioleoyl-sn-glycero-3-phosphocholine (DOPC), 1,2-dipalmitoyl-sn-glycero-3-phosphocholine (DPPC), and 1-palmitoyl-2-oleoyl-glycero-3-phosphocholine (POPC) were obtained from Avanti® Polar Lipids. Native α- or β-CD, and randomly methylated α- or β-CD were purchased from Fisher Scientific. Mono-6-O-(p-toluenesulfonyl)-CDs were obtained from Tokyo Chemical Industry Co., Ltd. Mono-6-Iodo-6-deoxy-β-CD was purchased from AraChem. Synthetic chemicals and solvents were purchased from Sigma-Aldrich, Tokyo Chemical Industry Co., Ltd, or Thermo Fisher Scientific and used without further purification unless otherwise specified. Thin-layer chromatography (TLC) was carried out on E. Merck silica gel 60 F254 analytical plates. The detection and visualization of cyclodextrin derivatives were performed by dipping the plates in Phosphomolybdic Acid solution or 20% sulfuric acid-ethanol followed by heating.

b) Compound characterization

^{1}H and ^{13}C NMR spectra were recorded on Bruker AVA-300 or Varian VX-500 MHz. Chemical shifts were reported in parts per million (δ) and calibrated using internal tetramethylsilane (TMS) standard or with the reference relative to TMS for ^{1}H NMR spectra ($CDCl_3$ 7.26 ppm; D_2O 4.79 ppm) and for ^{13}C NMR spectra ($CDCl_3$ 77.16 ppm; CH_3 of acetone in D_2O 30.89 ppm). Multiplicity was indicated as follows: s (singlet); d (doublet); t (triplet); q (quartet); quin (quintet); m (multiplet); dd (doublet of doublet); td (triplet of doublet); brs (broad singlet) etc. Coupling constants were reported in Hz. HPLC analysis was carried out on an Eclipse Plus C8 analytical column with Phase A/Phase B gradients [Phase A: H_2O with 0.1% formic acid; Phase B: MeOH with 0.1% formic acid or Phase A: H_2O with 0.1% trifluoroacetic acid; Phase B: Acetonitrile with 0.1% trifluoroacetic acid] Electrospray Ionization-Time of Flight (ESI-TOF) spectra were obtained on an Agilent 6230 Accurate-Mass TOFMS mass spectrometer.

c) Fluorescence measurement and phase contrast imaging

Fluorescence measurements were carried out on a Tecan infinite F200 plate reader instrument. Phase-contrast microscopy was performed on Olympus BX51 microscope.

2. Experimental Procedure

a) Evaluation of lipase mimetic activity for modified CDs using a fluorogenic probe (compound **2**).

A corresponding CD derivative (**A1–A6** and **B1–B6**) was dissolved in 1 mL of 0.02 M bicine buffer solution (pH 8.0) to generate 1 mM CD mixture. Then, 10 uL of 100 mM DMSO solution of compound **2** was added to the reaction mixture and stirred immediately at 37 °C. Aliquots (20 uL) taken at the specified time points were subjected to plate reader to record the fluorescence intensity of product (λ_{ex}: 480 nm, λ_{em}: 525 nm). The percent yields of the reactions were obtained by comparing the fluorescence intensity of 1 mM of compound **1**.

b) General procedure for monitoring a reaction between GPLs and **A6**

A corresponding GPL dissolved in 50 μL of chloroform was added to a 2 mL glass vial followed by blowing a stream of argon gas to form a thin lipid film. The vial was further dried under the high vacuum for 1 h. Then, **A6** (or nucleophiles, for figure 4A) was added to the vials, followed by an addition of 600 μL solution of pH 8.0 bicine buffer (0.02 M). The reaction concentrations of GPLs and **A6** were 1 and 4 mM, respectively. The reaction mixture was stirred at 750 rpm using a magnetic stirring bar at a given temperature (37 or 50 °C) for 3 days. The resulting solution was vortexted and 20 μL of reaction sample was transferred to a 0.6 mL microcentrifuge tube, and diluted with 200 μL of methanol containing 1N HCl (2% v/v). After centrifuged at 800 rpm for 1 min, 150 μL of supernatant was collected and subjected to HPLC/ELSD/MS analysis.

c) Imaging analysis of vesicular structures

A corresponding GPL dissolved in 50 μL of chloroform was added to a 2 mL glass vial followed by blowing a stream of argon gas to form a thin lipid film. The vial was further dried under the high vacuum for 1 h. Then, 600 μL solution of pH 8.0 bicine buffer (0.02 M) was added to the vial and stirred at 750 rpm, with or without 4 mM of **A6**. After stirring at 37° C for 3 days, 2 μL of a corresponding sample was analyzed by phase-contrast microscopy to determine the presence of vesicle structures.

3. Synthetic Procedure and Characterization of New Compounds

Mono-NH_2-CDs (**A5** and **B5**)[1] and per-NH_2-CDs (**A6** and **B6**)[2] were prepared in accordance with reported synthetic methods.

Compound 2: Compound **2** was prepared through a modified synthetic procedure.[3] Palmitoyl chloride (201 μL, 0.662 mmol) was added dropwise to a mixture of fluorescein (0.20 g, 0.60 mmol), sodium hydride (52.5 mg, 1.2 mmol) and $ZnCl_2$ (20 mg, 0.15 mmol) in anhydrous *N*,*N*-dimethylformamide (10 mL) at 0 °C. After 12 h stirring at room temperature, a little piece of ice was added to quench residual sodium hydride. The mixture was then washed with brine, extracted with EtOAc, dried over anhydrous Na_2SO_4, and concentrated. The crude product was purified by silica-gel flash column chromatography (Hexane:EtOAc = 3:1) to afford compound **2** (110 mg, 32.0 % yield) as a yellow solid; ^1H NMR ($CDCl_3$, 500 MHz) δ 7.96 (d, *J* = 7.3 Hz, 1H), 7.65–7.50 (m, 2H), 7.05 (d, *J* = 7.1 Hz, 1H), 7.02–6.94 (m, 1H), 6.76–6.66 (m, 2H), 6.66–6.60 (m, 1H), 6.56 (d, *J* = 8.6 Hz, 1H), 6.46 (dd, *J* = 8.7, 2.1 Hz, 1H), 5.60 (brs, 1H), 2.50 (t, *J* = 7.5 Hz, 2H), 1.68 (quin, *J* = 7.3 Hz, 2H), 1.35–1.15 (m, 24H), 0.81 (t, *J* = 6.7 Hz, 3H); ^{13}C NMR ($CDCl_3$, 125 MHz) δ 172.3, 169.8, 157.9, 153.1, 152.3, 152.1, 151.9, 135.4, 130.0, 129.5, 129.2, 126.5, 125.2, 124.2, 117.6, 116.6, 112.6, 111.1, 110.5, 103.2, 82.8, 34.5, 32.1, 29.8, 29.8, 29.7, 29.6, 29.5, 29.4, 29.2, 25.0, 22.9, 14.3; HRMS (ESI) m/z calcd for $C_{36}H_{43}O_6$ $[M+H]^+$: 571.3054, found: 571.3054.

Cyclodextrin A3: A mixture of mono-6-Iodo-6-deoxy-α-CD (45.6 mg, 0.042 mmol)[4] and imidazole (28.7 mg, 0.42 mmol) in anhydrous *N*,*N*-dimethylformamide (0.8 mL) was stirred at 80 °C for 20 h. After cooling down to room temperature, a large volume of acetone was added to generate white precipitate. Resulting solid was filtered and thoroughly washed with acetone to yield **A3** (33.3 mg, 77.3% yield) as a white solid; ^1H NMR (D_2O, 500 MHz) δ 7.77 (brs, 1H), 7.20 (brs, 1H), 7.01 (brs, 1H), 5.19–4.90 (m, 6H), 4.61–4.46 (d, *J* = 14.7 Hz, 2H), 4.23 (dd, *J* = 13.9, 7.8 Hz, 2H), 4.08–3.69 (m, 17H), 3.66–3.43 (m, 10H), 3.40–3.19 (m, 3H), 3.04 (d, *J* = 11.7 Hz, 2H); ^{13}C NMR (D_2O, 125 MHz) δ 138.2, 127.1, 121.0 (Imidazole), 101.3, 101.2, 100.9 (Cyclodextrin C1), 82.8, 81.3, 81.1, 81.0, 80.7 (C4), 73.1, 73.1, 72.9, 72.9, 72.4, 71.9, 71.8, 71.6, 71.5, 71.4, 70.7 (C2, C3, C5), 60.5, 60.2, 60.1, 59.2 (C6), 47.6 (C6 with N attached); HRMS (ESI) m/z calcd for $C_{39}H_{63}N_2O_{29}$ $[M+H]^+$: 1023.3511, found: 1023.3501.

Cyclodextrin B3: A mixture of mono-6-Iodo-6-deoxy-β-CD (93.6 mg, 0.075 mmol) and imidazole (25.6 mg, 0.375 mmol) in anhydrous *N*,*N*-dimethylformamide (0.7 mL) was stirred at 70 °C for 3 days. After cooling down to room temperature, a large volume of acetone was added to generate white precipitate. Resulting solid was filtered and recrystallization was

carried out by using an acetone-water pair to yield **B3** (74 mg, 83% yield) as a white solid; ^{1}H NMR (D_2O, 500 MHz) δ 7.78 (brs, 1H), 7.18 (s, 1H), 7.02 (s, 1H), 5.10–4.90 (m, 7H), 4.54 (d, *J* = 14.2 Hz, 2H), 4.20 (dd, *J* = 14.7, 8.6 Hz, 2H), 4.03 (t, *J* = 9.0 Hz, 1H), 3.97–3.75 (m, 21H), 3.68–3.44 (m, 13H), 3.37 (t, *J* = 9.4 Hz, 1H), 3.23 (d, *J* = 12.5 Hz, 1H), 3.03–2.93 (m, 1H); ^{13}C NMR (D_2O, 125 MHz) δ 138.9, 127.6, 121.7 (imidazole), 102.5, 102.0 (Cyclodextrin C1), 83.7, 82.1, 81.8, 81.7, 81.3 (C4), 73.7, 73.5, 72.9, 72.7, 72.6, 72.5, 72.4, 72.3, 72.2, 71.5 (C2, C3, C5), 61.2, 60.9, 60.0 (C6), 48.5 (C6 with N attached); HRMS (ESI) m/z calcd for $C_{45}H_{73}N_2O_{34}$ $[M+H]^{+}$: 1185.4039, found: 1185.4031.

Cyclodextrin B4: A mixture of per-6-bromo-6-deoxy-β-CD (150 mg, 0.095 mmol)[5] and imidazole (324 mg, 4.76 mmol) in anhydrous *N*,*N*-dimethylformamide (1 mL) was stirred at 80 °C for 3 days. After cooling down to room temperature, a large volume of acetone was added to generate white precipitate. The resulting precipitate was filtered and thoroughly washed with ethanol to yield **B4** (130 mg, 92% yield) as a white solid; ^{1}H NMR (D_2O, 500 MHz) δ 8.77 (brs, 7H), 7.50 (brs, 7H), 7.37 (brs, 7H), 5.04 (brs, 7H), 4.56–4.34 (m, 14H), 4.15 (dd, *J* = 14.8, 3.5 Hz, 7H), 3.98 (td, *J* = 9.4, 2.2 Hz, 7H), 3.53 (m, 7H), 3.32 (td, *J* = 9.2, 1.5 Hz, 7H); ^{13}C NMR (D_2O, 125 MHz) δ 136.7, 123.7, 120.8, 102.6, 82.8, 72.7, 72.3, 70.0, 50.3; HRMS (ESI) m/z calcd for $C_{63}H_{87}N_{14}O_{28}$ $[M+3H]^{3+}$: 495.8599, found: 495.8592.

4. Supporting Figures

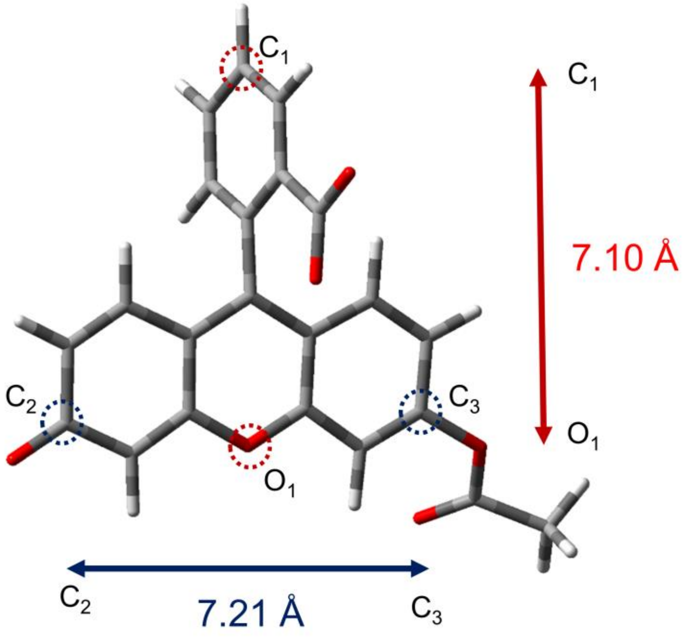

Fig. S1 Dimensions of an acylated fluorescein calculated by ground-state geometry optimization via density functional theory (DFT). (gaussian 09, B3LYP/6-31G*).

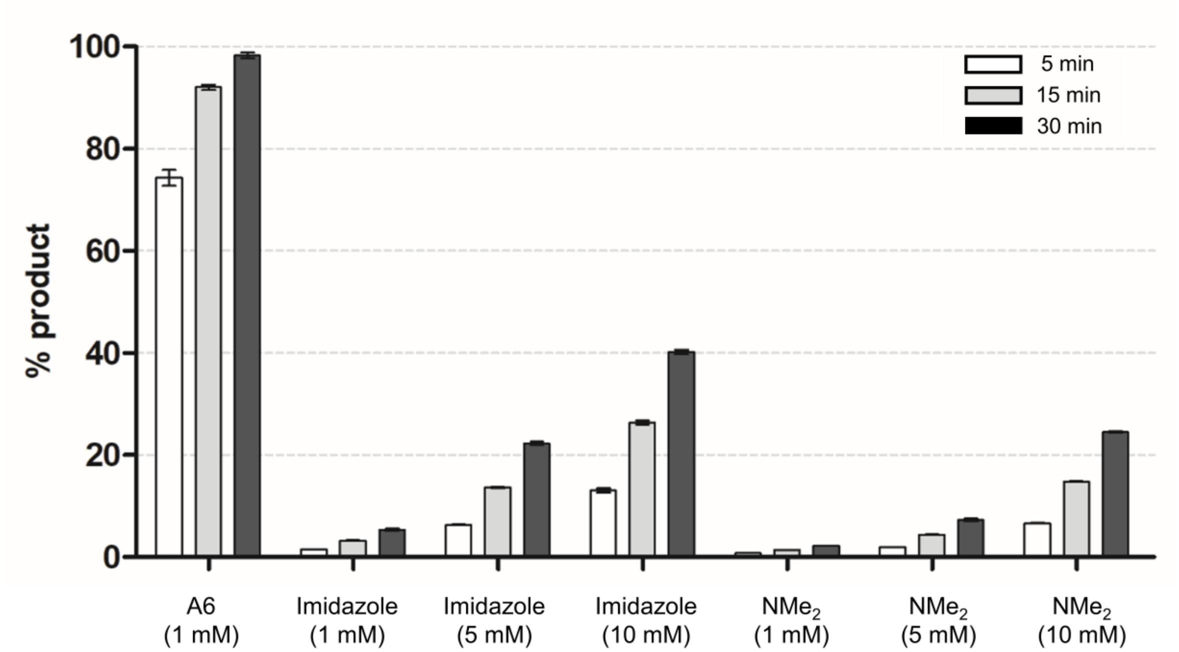

Fig. S2 The percent yield for compound **1** generated from the reaction between compound **2** (1 mM) and **A6** (1 mM) or 1, 5, and 10 mM of imidazole or dimethylamine, after 5, 15, and 30 min. Reactions were performed in 0.02 M bicine buffer (pH 8.0) with 1% DMSO (*v*/*v*) at 37 ˚C. Error bars represent standard deviation from n=3 replicates.

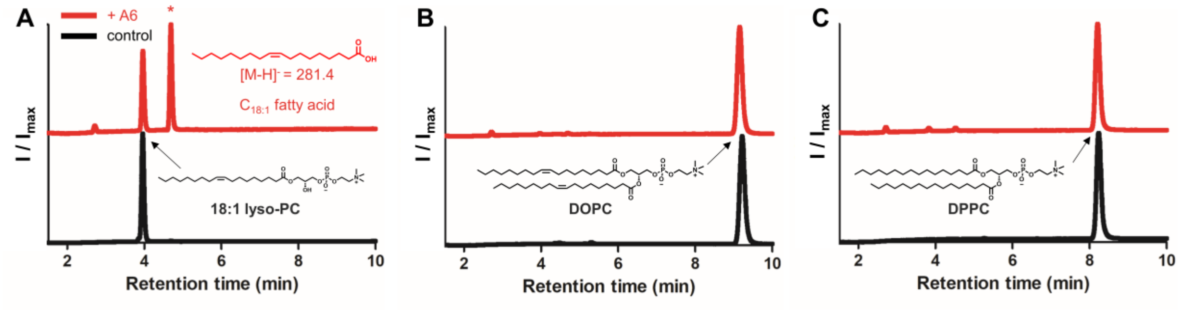

Fig. S3 Normalized HPLC-ELSD traces of the reaction between **A6** (4 mM) and (A) 18:1 lyso-PC (1 mM), (B) DOPC (1 mM), and (C) DPPC (1 mM) (upper red lines) or controls (lower black lines). All reactions were performed in 0.02 M bicine buffer (pH 8.0) at 37 ˚C for 3 days.

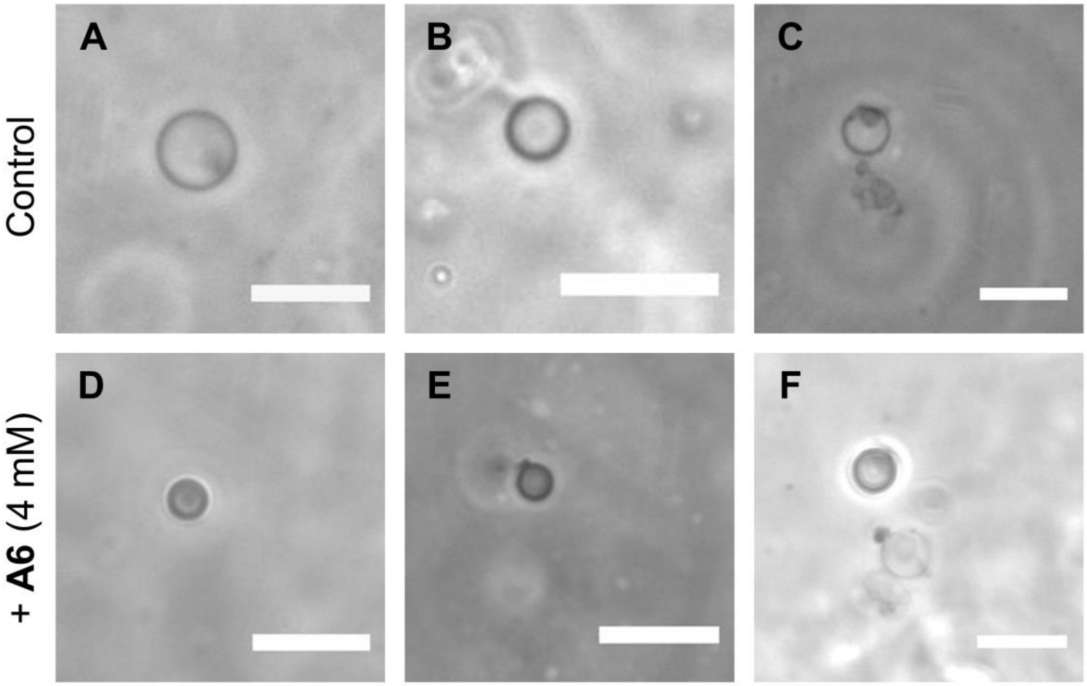

Fig. S4 Bright field images of vesicles from the self-assembly of DOPC (A,D), POPC (B,E), and DPPC (C,F). Upper images (A,B,C) were taken from control samples and lower images (D,E,F) were taken after the reaction between **A6** (4 mM) and corresponding lipids (1 mM each) in 0.02 M bicine buffer (pH 8.0) at 37 ˚C for 3 days. Scale bars denote 10 μM.

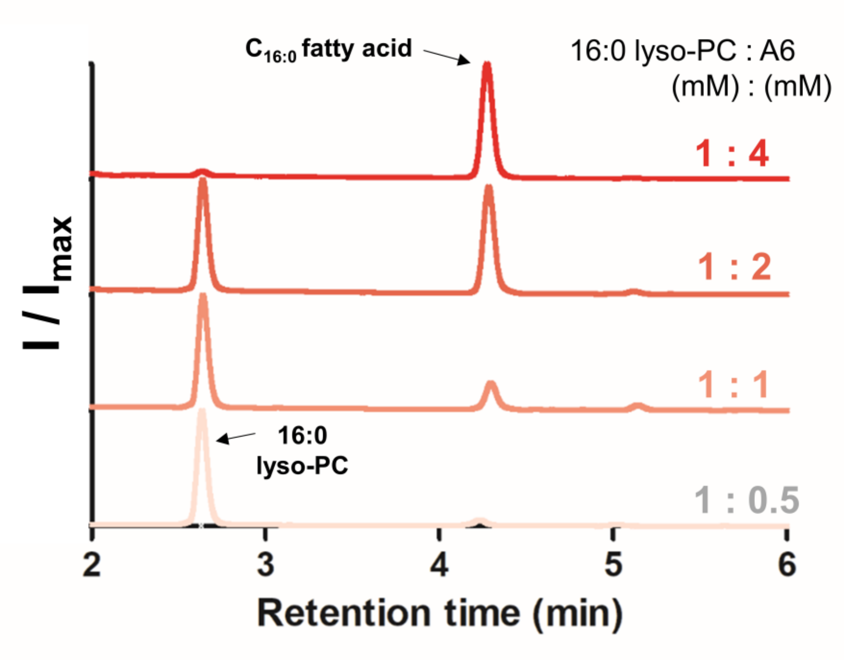

Fig. S5 Evaluation of lipid to CD ratio in the reaction between 16:0 lyso-PC and **A6**. Reactions were conducted in 0.02 M bicine buffer (pH 8.0) at 50 ˚C for 3 days.

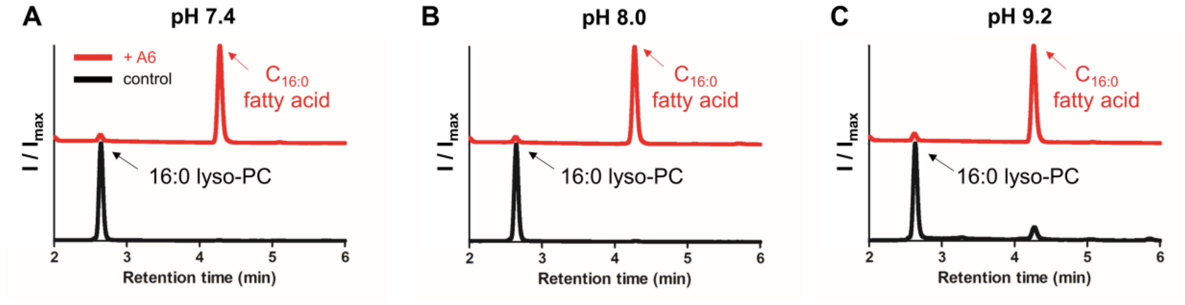

Fig. S6 Hydrolytic activity of **A6** in different pH buffers. Normalized HPLC-ELSD traces of the reaction between **A6** (4 mM) and 16:0 lyso-PC (1 mM) in (A) 0.02 M HEPES buffer at pH 7.4, (B) 0.02 M bicine buffer at pH 8.0, and (C) 0.02 M carbonate-bicarbonate buffer at pH 9.2. Reactions were performed at 50 ˚C for 3 days.

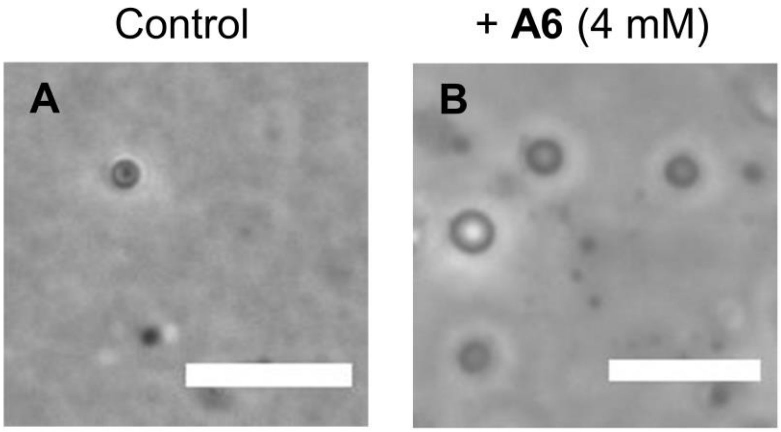

Fig. S7 Bright field images of vesicles from a combined mixture of DOPC, POPC, DPPC, 16:0 Lyso PC, and 18:1 lyso PC (1 mM each) (A) and after the reaction with **A6** (4 mM) in 0.02 M bicine buffer (pH 8.0) at 37 ˚C for 3 days (B). Scale bars denote 10 μM.

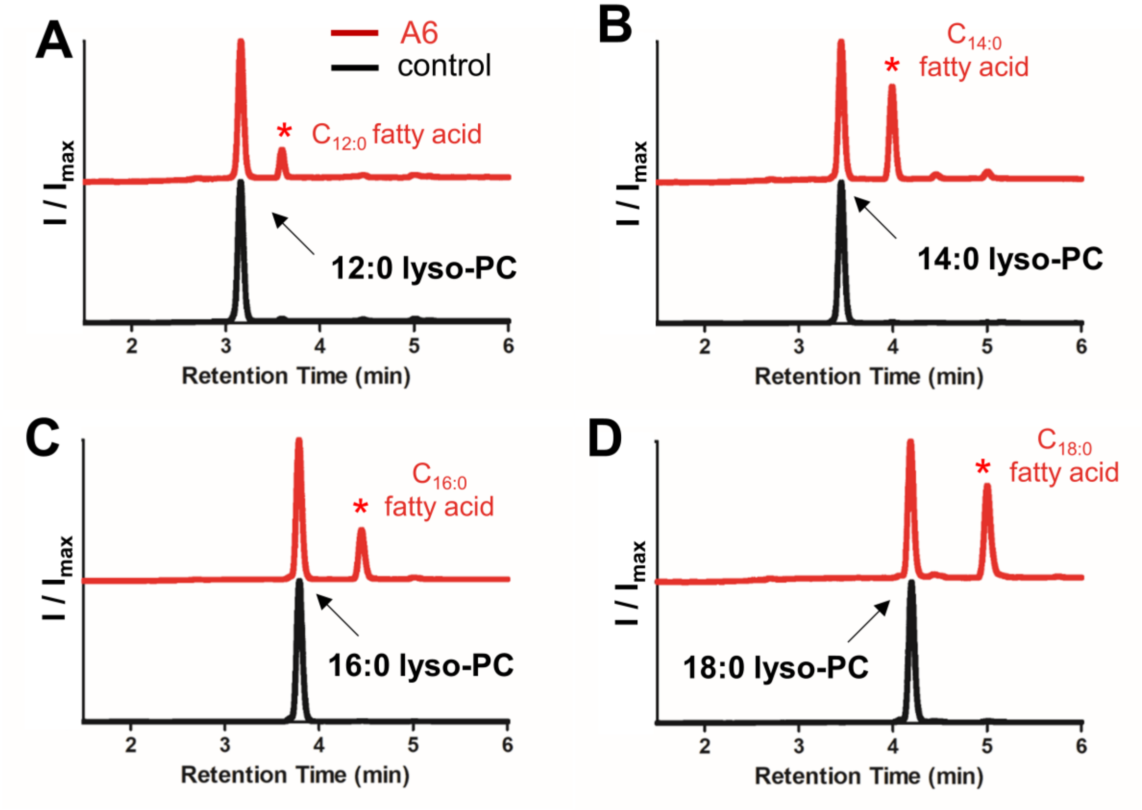

Fig. S8 Normalized HPLC-ELSD traces of the reaction between **A6** (1 mM) and (A) 12:0 lyso-PC (1 mM), (B) 14:0 lyso-PC (1 mM), (C) 16:0 lyso-PC (1 mM), and (D) 18:0 lyso-PC (1 mM) under 0.02 M bicine buffer (pH 8.0) at 50 ˚C for 3 days.

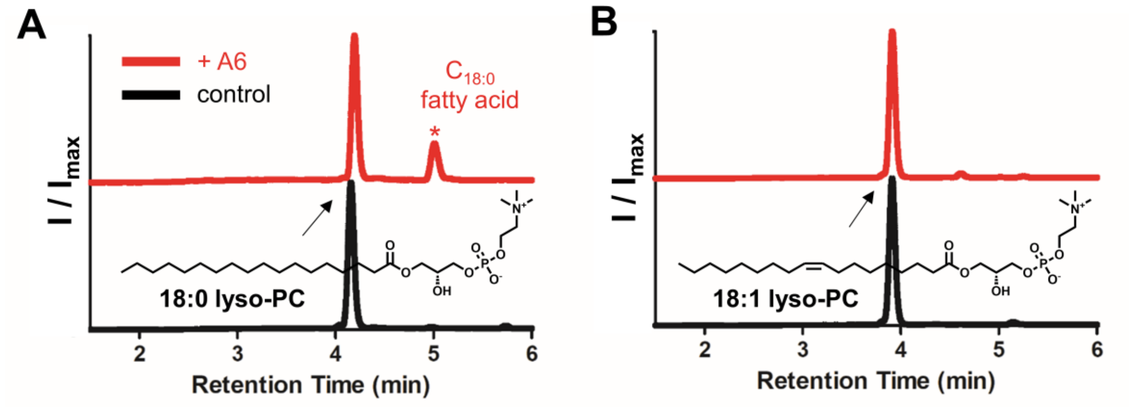

Fig. S9 Normalized HPLC-ELSD traces of the reaction between **A6** (1 mM) and (A) 18:0 lyso-PC (1 mM) and (B) 18:1 lyso-PC (1 mM) under 0.02 M bicine buffer (pH 8.0) at 37˚C for 3 days.

5. NMR spectra

Compound 2

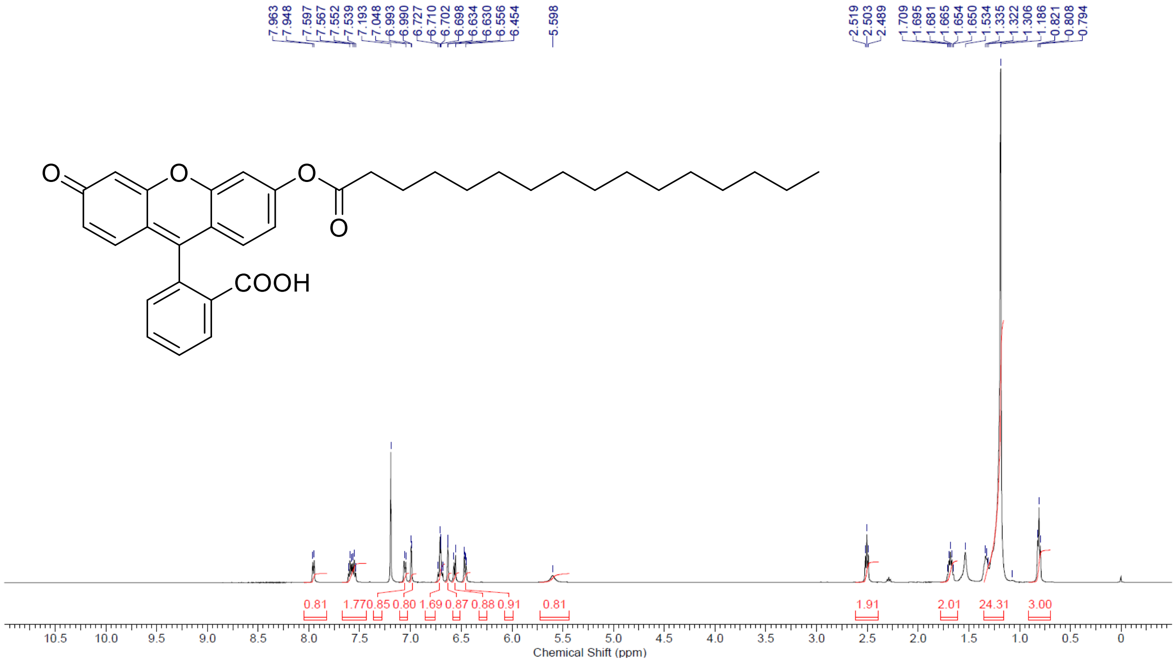

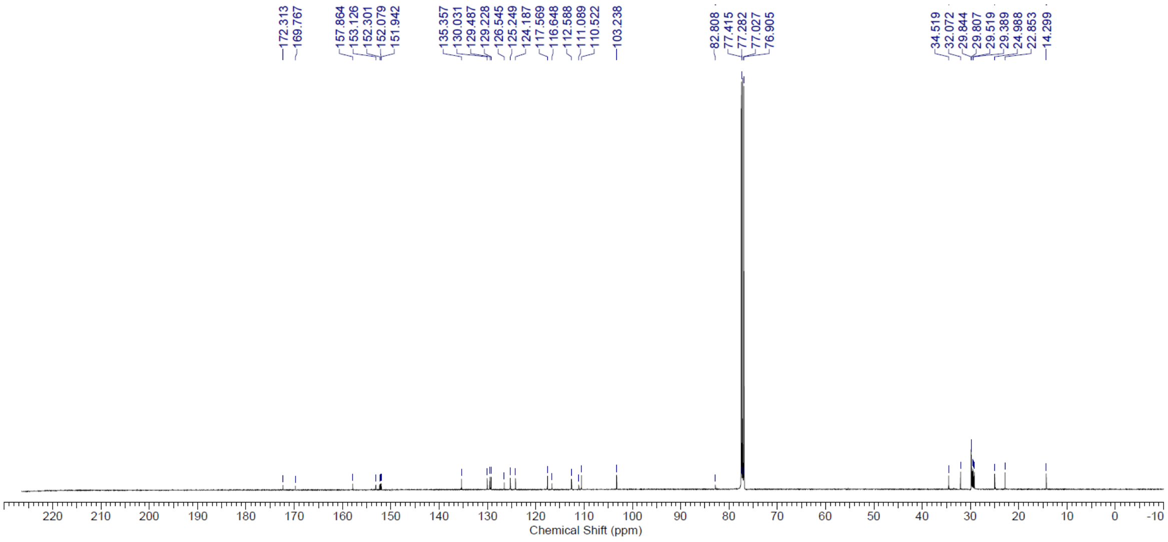

A3.

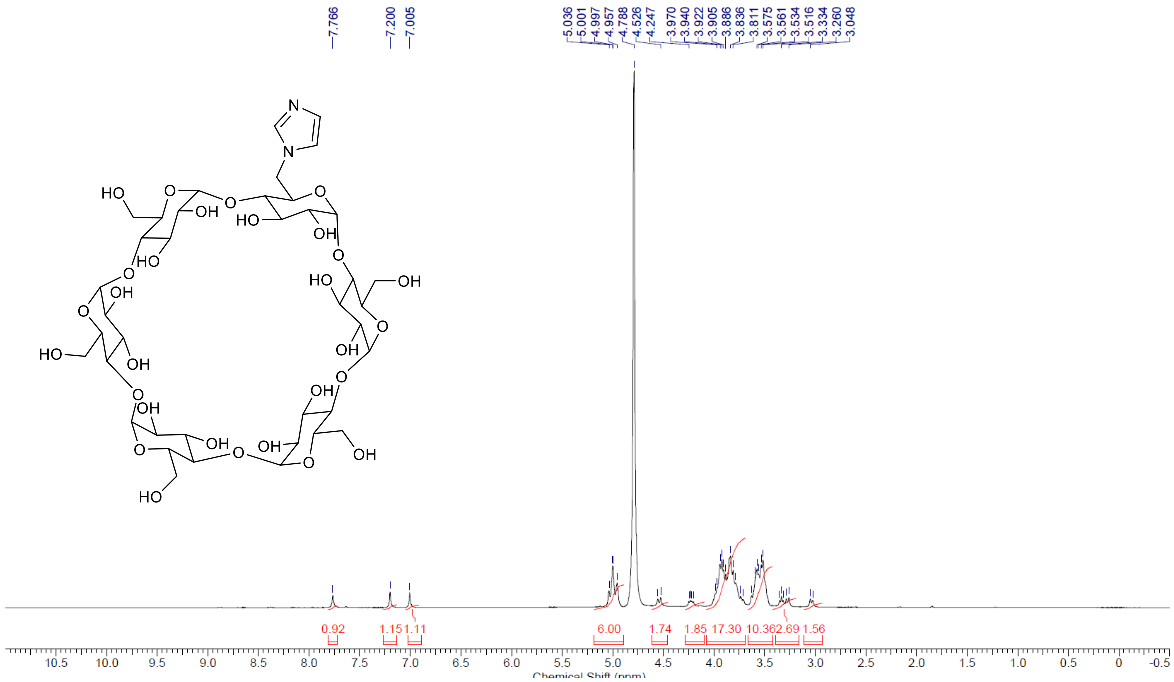
7.766
7.200
7.005
5.036
5.001
4.997
4.957
4.788
4.526
4.247
3.970
3.940
3.922
3.905
3.886
3.836
3.811
3.575
3.561
3.534
3.516
3.334
3.260
3.048
0.92
1.15
1.11
6.00
1.74
1.85
17.30
10.36
2.69
1.56
[illegible]

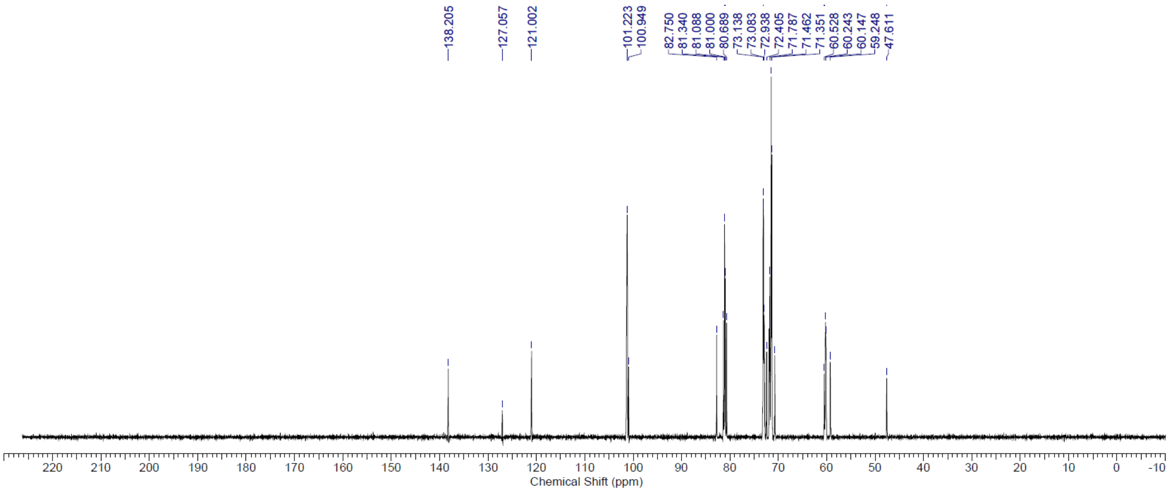
138.205
127.057
121.002
101.223
100.949
82.750
81.340
81.088
81.000
80.689
73.138
73.083
72.938
72.405
71.787
71.462
71.351
60.528
60.243
60.147
59.248
47.611
Chemical Shift (ppm)

B3.

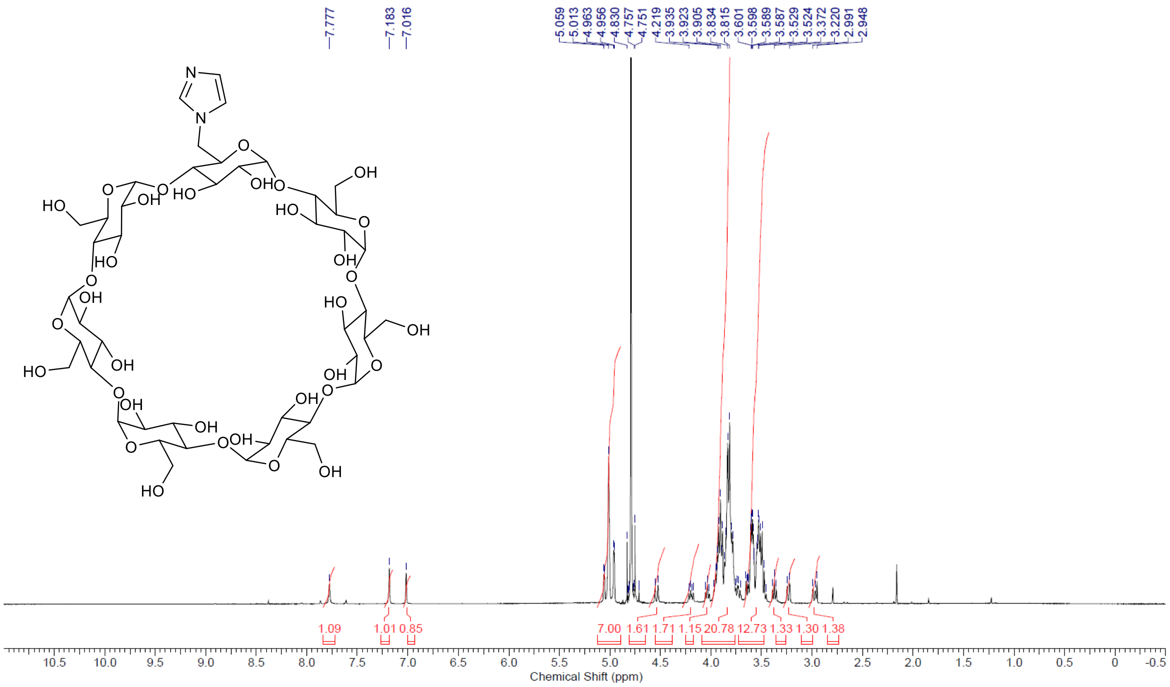

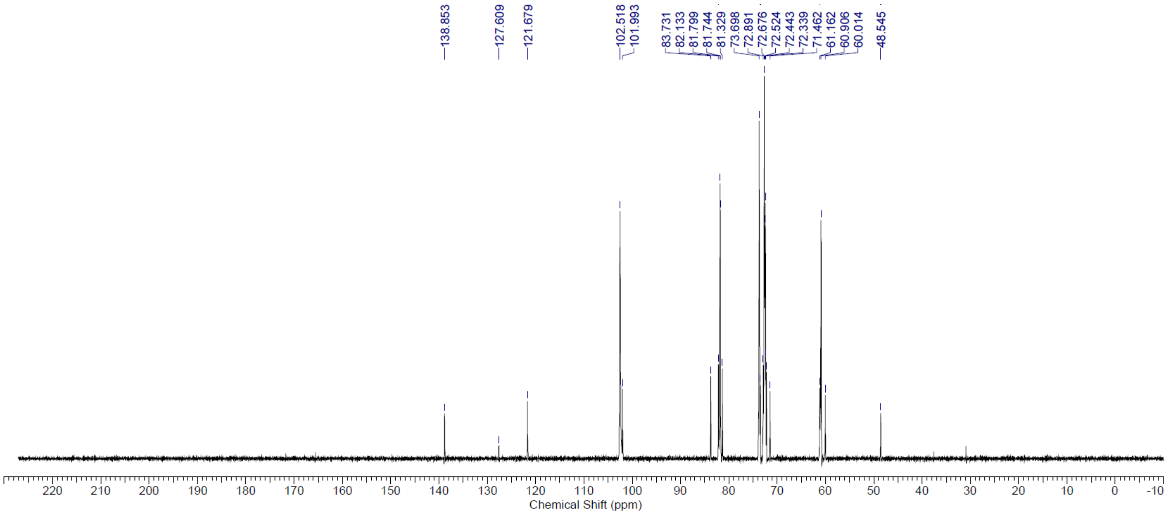

B4.

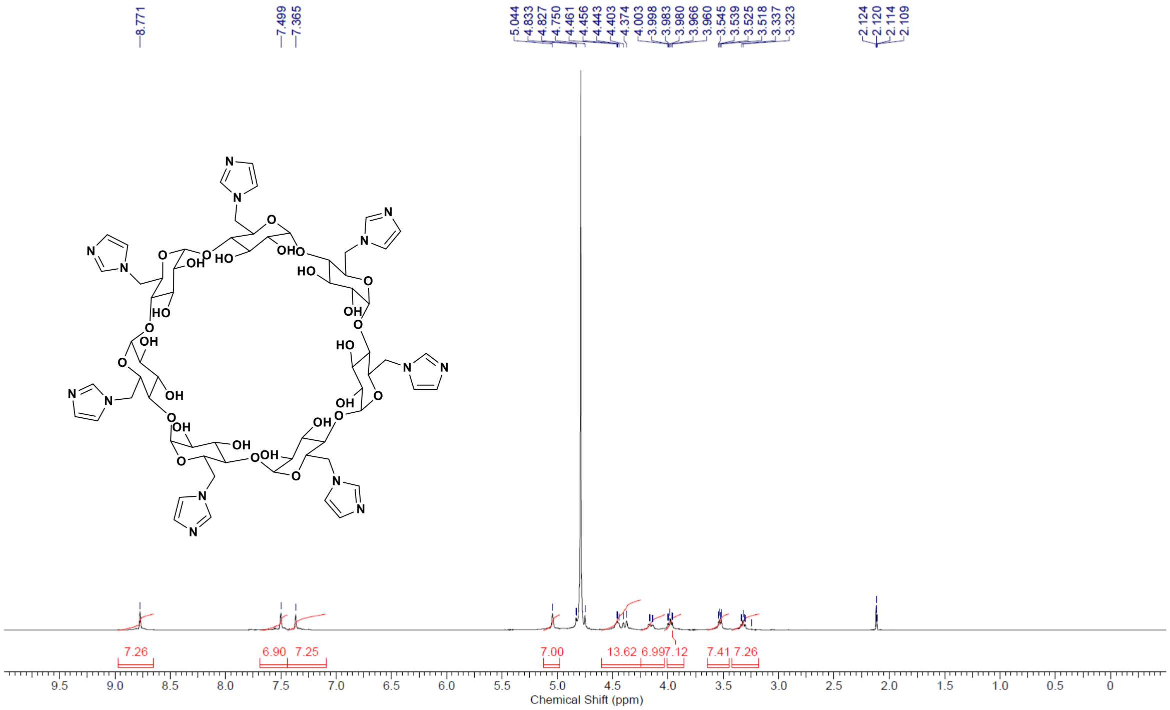

Chemical Shift (ppm)

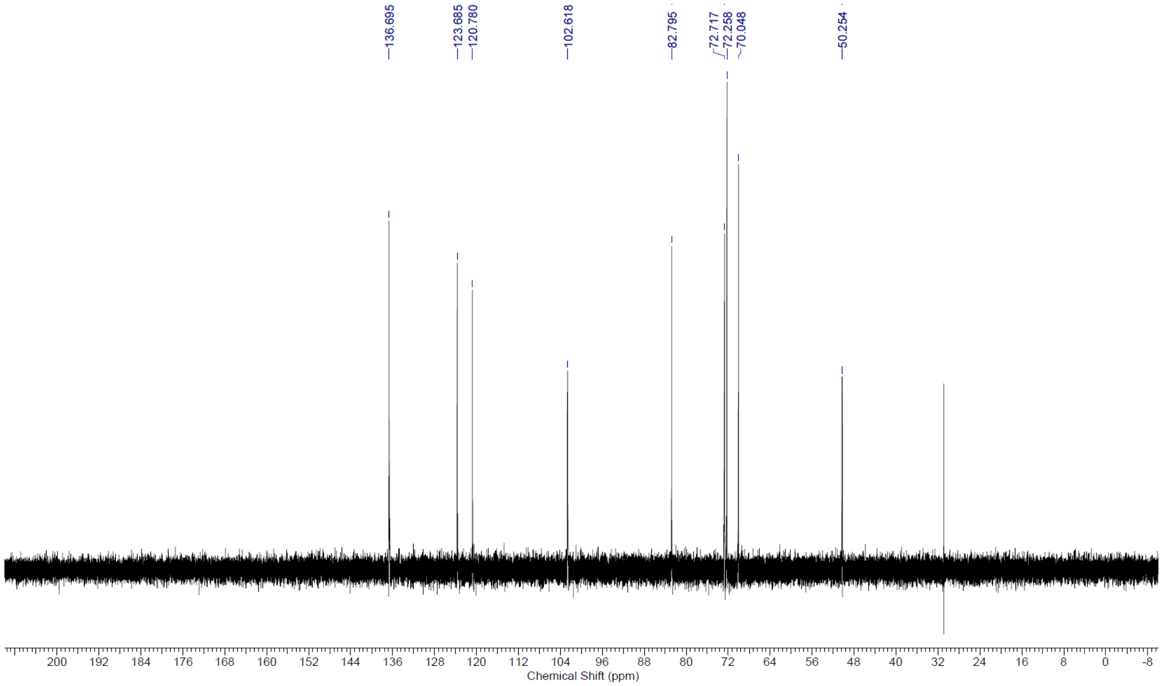

136.695
123.685
120.780
102.618
82.795
72.717
72.258
70.048
50.254
Chemical Shift (ppm)

6. References

1 W. Tang, S. C. Ng, *Nat. Protoc.* 2008, **3**, 691–697.

2 N. Mourtzis, K. Eliadou, C. Aggelidou, V. Sophianopoulou, I. M. Mavridis, K. Yannakopoulou, *Org. Biomol. Chem.* 2007, **5**, 125–131.

3 Y. Yang, P. Babiak, J. L. Reymond, *J. Helv. Chim. Acta* 2006, **89**, 404–415.

4 L. D. Melton, K. N. Slessor. *Carbohydr. Res.* 1971, **18**, 29–37.

5 D. Vizitiu, C. S. Walkinshaw, B. I. Gorin, G. R. J. Thatcher, *J. Org. Chem.* 1997, **62**, 8760–8766.
